# Cryostimulation for Post-exercise Recovery in Athletes: A Consensus and Position Paper

**DOI:** 10.3389/fspor.2021.688828

**Published:** 2021-11-24

**Authors:** Romain Bouzigon, Olivier Dupuy, Ivo Tiemessen, Massimo De Nardi, Jean-Pierre Bernard, Thibaud Mihailovic, Dimitri Theurot, Elzbieta Dorota Miller, Giovanni Lombardi, Benoit Michel Dugué

**Affiliations:** ^1^Université de Franche-Comté, UFR STAPS Besançon, Laboratoire C3S (EA4660), Axe Sport Performance, Besançon, France; ^2^Society Inside the Athletes 3.0, Sport Performance Optimization Complex (COPS25), Besançon, France; ^3^Society Aurore Concept, Noisiel, France; ^4^Université de Poitiers, Laboratoire MOVE (EA 6314), Faculté des Sciences du Sport, Poitiers, France; ^5^Ecole de Kinésiologie et des Sciences de l'Actvivité Physique (EKSAP), Faculté de Medecine, Université de Montreal, Montreal, QC, Canada; ^6^ProCcare BVBA, Antwerp, Belgium; ^7^Mobilito Sport, Amsterdam, Netherlands; ^8^Krioplanet Ltd, Treviglio, Italy; ^9^Department of Experimental Medicine, Università Degli Studi di Genova, Genoa, Italy; ^10^Air Liquide Group International Expert in Cryogenic Applications Cryolor, Ennery, France; ^11^Department of Neurological Rehabilitation, Medical University of Lodz, Lodz, Poland; ^12^Laboratory of Experimental Biochemistry and Molecular Biology, IRCCS Istituto Ortopedico Galeazzi, Milan, Italy; ^13^Department of Athletics, Strength and Conditioning, Poznań University of Physical Education, Poznań, Poland

**Keywords:** cryotherapy, cryostimulation, recovery, sport recovery, cold therapy, whole-body cryotherapy, whole-body cryostimulation

## Abstract

Recovery after exercise is a crucial key in preventing muscle injuries and in speeding up the processes to return to homeostasis level. There are several ways of developing a recovery strategy with the use of different kinds of traditional and up-to-date techniques. The use of cold has traditionally been used after physical exercise for recovery purposes. In recent years, the use of whole-body cryotherapy/cryostimulation (WBC; an extreme cold stimulation lasting 1–4 min and given in a cold room at a temperature comprised from −60 to −195°C) has been tremendously increased for such purposes. However, there are controversies about the benefits that the use of this technique may provide. Therefore, the main objectives of this paper are to describe what is whole body cryotherapy/cryostimulation, review and debate the benefits that its use may provide, present practical considerations and applications, and emphasize the need of customization depending on the context, the purpose, and the subject's characteristics. This review is written by international experts from the working group on WBC from the International Institute of Refrigeration.

## Introduction

Concerning sport competition and training, a well-organized training program combined with appropriate recovery strategies are keys for success. Different kinds of strategies for enhancing recovery and recovery capacities have been developed and quite recently, we presented a series of meta-analysis dealing with the use of different recovery techniques after physical exercise (training and/or competition) to reduce markers of muscle damage, soreness, fatigue, and inflammation. In addition, we compared the effects of one strategy versus another (Dupuy et al., 2018). Among the reviewed recovery techniques, whole-body cryotherapy or whole-body cryostimulation (WBC) was shown to positively impact the recovery.

Cold exposure (locally or whole-body) has been used for a very long time in the context of sports and medicine to relieve pain and inflammatory symptoms through cold-induced analgesia. Nowadays, WBC is defined as extreme cold therapy or stimulation which is applied by placing a subject in a cold room, for 1–3 or 4 min, where the air temperature can reach extremely low values (felt temperature ranging from −60 to −195°C). Individuals are exposed with minimal clothing with protections on the feet, hands, and ears. A small surgical mask is applied to protect the airways. An alternative is the use of partial-body cryotherapy/cryostimulation (PBC) where the subject's body is exposed in a cabin but the head is not exposed (Bouzigon et al., 2016).

The main effect of WBC is analgesia, related to the impact of very-low temperatures on the nervous system disabling the functional connections with the sensory receptors and proprioceptors conduction in sensory fibers. This process is responsible for the reduction of increased muscle tension. The use of WBC/PBC as cold therapy reduces post-traumatic edema, inflammation (facilitates the exchange of inflammatory products, carbon dioxide, accelerates metabolism, provides nutritional substrates, molecular oxygen, etc.), and facilitates post-exercise recovery by reducing the sensations of delayed onset muscle soreness (DOMS) and exercise-induced muscle damage (EIMD; Dupuy et al., 2018).

This paper aimed to provide general information on WBC and PBC when it is used after physical exercise. The technologies that are regularly used are described and a specific focus is made on the potential recovery benefits that athletes may benefit from following exercise and physical activity.

## Cryostimulation Definitions (Protocol and Technologies)

**Figure 1:**
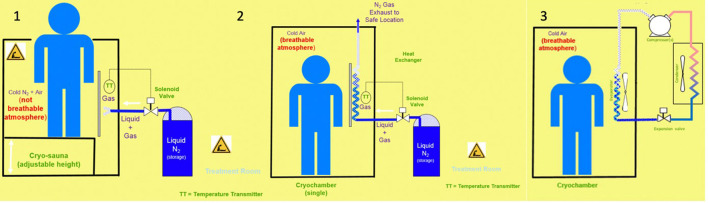
1: Cryosauna: Principle with liquid N2/direct injection/PBC/individual. 2: Cryochamber: Principle with liquid N2/indirect injection/WBC/individual or collective. 3: Cryochamber: Principle with mechanical refrigeration (single stage)/WBC/collective.

Cryostimulation is a relatively new technique consisting on exposing a part of or the whole-body to extreme cold for a short period of time. Based on the positive effects of cold immersion used since the antiquity, cryostimulation is an innovative technology that is developing significantly (Bouzigon et al., 2016). The two types of technologies are available in the market, WBC and PBC. The WBC chambers, named cryochambers, are composed of 1–3 rooms in which the individual stays between 1 and 4 min and where the whole-body is exposed. The two main cold production methods that can be used are: mechanical refrigeration based on vapor compression cycles with refrigerants and/or cryogenic fluids, mainly liquid nitrogen, as it is a widely used and common fluid in many industries (Figure 1). There are two types of WBC chambers: static cold chambers and forced convection chambers based on wind chill. Cryochambers are more complex devices, but they avoid anoxia issues by using indirect injections in the chambers and the whole body is in a cold environment for the treatment. PBC tanks, named cryosaunas, are adapted for single usage for an individual, where cryogenic fluid is injected and vaporized around the body. However, the head must stay above the gaseous environment to preserve breathing (Dugué et al., 2020).

The cryostimulation devices provide the cold temperatures. The manufacturers state that temperatures range from −110 to −195°C in cryosaunas, from −60 to −160°C in static cold WBC technologies and from −40 to −60°C in forced convection WBC technologies (Bouzigon et al., 2016, 2017). Temperatures recorded inside the different devices during an exposure actually range from −10 to −42°C in cryosaunas and −34°C in a forced convection WBC chamber (Savic et al., 2013; Bouzigon et al., 2019). The temperatures in a static cold WBC chamber during the exposure are not shown in the literature.

Mean skin temperature variation after a 3-min exposure is ~-8°C in a cryosauna (mean skin temperature after exposure between 22 to 24°C); ~-11°C in a static cold WBC chamber (mean skin temperature after exposure between 18 to 20°C); and ~-14°C in a forced convection WBC chamber (mean skin temperature after exposure between 16 to 18°C; Bouzigon et al., 2016, 2019). The exposure time ranges from 1 to 4 min (Bouzigon et al., 2016). The exposure duration depends on the protocol used but could be the same between the different devices. The 1-min exposure protocol is commonly used for the first exposure in a non-initiated individual.

## General Physiological Reactions After WBC/PBC

Humans have a constant internal core temperature of ~37°C. To maintain such internal temperature, the body needs to thermoregulate (Moellering and Smith, 2012). Thermoregulation is a complex physiological process, which is not entirely understood. A great part of the total body energy is produced as heat to maintain the internal temperature in our organism (Stocks et al., 2004). During cold stress, changes in the endocrine, circulatory, neuromuscular, and immunological systems occurred (Kellogg, 2006). The key point in the thermoregulation is cutaneous circulation. The dermis is characterized by the presence of a higher number of cold receptors (10-fold higher) in comparison to heat receptors.

The current view is the following after a WBC/PBC exposure: vasoconstriction that is induced after cold exposure may play a role in decreasing the amount of blood in and around the muscles and in some organs (Charkoudian, 2003), in decreasing the cell permeability and leaking, in diminishing the fluid diffusion in interstitial space that can occur after physical exercise to reduce edema development, and in reducing inflammation (Banfi et al., 2009b). In the context of recovery after physical exercise, a WBC/PBC exposure induces a lower muscle temperature that may decrease muscle enzyme activities, metabolism, inflammation, and secondary degradation after hypoxia (lowering ischemia/reperfusion problems) that may help recovery (Bouzigon et al., 2016). The effects of the WBC/PBC on physical recovery are discussed in a paragraph below (Section WBC—PBC Can Diminish Inflammation and Induce Analgesic Effects). During vasoconstriction, there is a strong activation of the sympathetic system accompanied with the release of noradrenalin. Noradrenalin is known to have an impact on pain, and this may explain how WBC can contribute in relieving the pain symptoms. Other compounds, such as endorphins may have an impact. Besides such mechanisms, cryogenic temperature reduces the conduction velocity of sensory nerve fibers and impulsion in the slow conducting C fibers disabling the sensory receptors as well as their connections with the proprioceptors. In addition, it seems that there is a decrease in the production of pro-inflammatory and oxidative substances whereas the anti-inflammatory and anti-oxidative compounds are produced in larger quantities (Banfi et al., 2010; Lombardi et al., 2017).

After exposure, cold-induced vasodilation occurs, enabling a four-fold higher blood flow than normal. Such a situation is observed about 4 min after the contact with cryogenic temperatures. Moreover, this robust blood circulation change can last a few hours following exercise, resulting in the elimination of metabolic products. It is estimated that the return to basal skin temperatures is likely after 14 min. At distance from the cold stimulation, an increase in the parasympathetic cardiac control may also happen (Louis et al., 2020) even during the night (Douzi et al., 2018). Such changes related to the central nervous system may have impacts on the psyche where lower fatigue sensation and mood improvement have been noted with possible positive impacts on clinical depression syndromes, and improvement in sleep quality (Rymaszewska et al., 2008). However, there are a range of individual responses to cold due to inter-individual differences, such as body size, fitness level, amount of subcutaneous fat, and gender (Miller et al., 2012).

### WBC—PBC Can Diminish Inflammation and Induce Analgesic Effects

The main aim of cryostimulation is represented by the anti-inflammatory and analgesic effects. These effects, although proven and reported by several research, are highly variable and there is still no consensus on the reliability of the outcomes (Bouzigon et al., 2016; Lombardi et al., 2017). This is clearly evidenced by the fact that participants who underwent cycles of cryostimulation almost invariably reported an improved sense of well-being feeling, accompanied with the improvement of inflammatory symptoms and pain (when present) (Lombardi et al., 2017). Several studies have tried to provide mechanistic explanations for this phenomenon by measuring clinically relevant changes in certain blood biomarkers of inflammation [e.g., interleukin (IL)-6, IL-1, IL-1 receptor antagonist (IL-1ra), IL-10, IL-4, tumor necrosis factor (TNF)-α, and C-reactive protein (CRP)] (Lombardi et al., 2017).

There are two published randomized controlled trials evaluating the effects of WBC on post-exercise recovery in physically active subjects. In a cross-over study by Hausswirth et al. (2011), well-trained runners who underwent three separate simulated races to induce muscle damage, followed by the three sessions (0, 24, and 48 h) of three different recovery strategies (passive, far-infrared therapy, and WBC). WBC was performed in a three-room device at −10, −60, and −110°C, respectively; the subjects entered the warmer rooms and remained in the therapy room for 3 min. Despite the similar kinetics of the muscle damage marker creatine phosphokinase (CK), a better and faster improvement of perceived pain, tiredness, and well-being following WBC compared with the other recovery strategies were reported (Hausswirth et al., 2011). In parallel, Selfe et al. (2014) failed to record any change in IL-6, regardless of the duration of the WBC exposure (1, 2, or 3 min, at −135°C). In this study, each WBC exposure consisted of 30 s precooling at −60°C followed by a randomized exposure for either 1, 2, or 3 min at −135°C.

However, several published reports demonstrated the anti-inflammatory effects of WBC. Tennis players exposed to WBC (20–30 s at −60°C and 3 min at −120°C) twice a day over 5 consecutive days during a training camp showed decreased circulating TNFα and increased circulating IL-6 in the treated athletes more than in the untreated athletes (60 and 30%, respectively). The blood samplings were performed 3 days before and 2 days after the camp (Ziemann et al., 2012). In 18 professional male volleyball players, WBC treatment (10–20 s at −60°C, 100–110 s at −120°C) performed before a session of submaximal exercise prevented exercise-induced rise of IL-1β and IL-6 that, in the absence of any treatment, attested about +60%. The setting expected two blood sampling, before and after the physical effort (Mila-Kierzenkowska et al., 2013). In healthy young men, IL-6 increased after a single session of WBC (as recorded after 30 min and 24 h) although less consistently, after 10 days of the treatment (Lubkowska et al., 2010). The WBC was performed by exposing subjects for 30 s at −60°C and 2.5 min at −130°C; baseline blood samples were taken in the morning before the first WBC session while the post-treatment sampling was performed in morning after the last WBC session. In a recent meta-analysis, Dupuy et al. (2018) reported that acute cryostimulation increased plasmatic IL-6.

The fact that a change in IL-6, either toward increase or decrease, is considered beneficial or not depends on the coexistence of both pro- and anti-inflammatory roles of this cytokine depending on the site and kinetics of secretion (i.e., liver-derived, chronically slightly elevated IL-6 acts in a pro-inflammatory fashion; skeletal muscle-derived, and temporarily limited peaked IL-6 acts in an anti-inflammatory fashion; Lombardi et al., 2016). Furthermore, in a group of volleyball players who underwent a 2-week training program, daily WBC led to an improvement in the growth factor profile [brain-derived neurotrophic factor (BDNF) and insulin-like growth factor 1 (IGF-1)] that may sustain muscle regeneration and limit the deterioration of physical performances (Jaworska et al., 2018). However, in elite athletes subjected to randomized WBC (3 min, −120°C, two times a day for 7 days) or no treatment, despite the significant reduction of reactive oxygen and nitrogen species (H_2_O_2_ and NO) and the concentrations of the pro-inflammatory mediators IL-1β and CRP, cryostimulation caused the decrease in IGF-1, BDNF, hepatocyte growth factor (HGF), platelet-derived growth factor (PDGFBB), and vascular endothelial growth factor (VEGF; Zembron-Lacny et al., 2020). These findings further suggest that WBC may act as an exercise-mimetic treatment, possibly associated to the cold-induced muscle contraction, stimulating the expression of those myokines whose release are typical following an acute bout of physical activity.

From a cellular point of view, the leukocytes were mostly unaffected by the WBC treatment in a group of professional rugby players (Lombardi et al., 2013) and in professional kayakers (Sutkowy et al., 2014), who underwent two-daily sessions of cryostimulation, during a training camp. On the contrary, Szygula and colleagues (Szygula et al., 2014) recorded an increase in leukocyte count during the first 20 sessions of WBC in students of a military academy and a return to baseline at the 30th session. Similarly, Ziemann et al. (2012) found an increase in whole leukocyte counts but not in the neutrophil and lymphocytes subpopulations in the tennis players. Noteworthy, when recorded, the changes in leukocyte count remained always within the physiological range. Consequently, mobilization, rather than generation, may explain this phenomenon although the biological significance remains unknown. The platelets, however remained mostly unaffected by the WBC treatment (Lombardi et al., 2013; Szygula et al., 2014; Ziemann et al., 2014), contrarily to what was observed in other situations of cold exposure as described in the case of winter swimming (Lombardi et al., 2011). These variable results may be due to the differences in the applied protocols as well as in the differences in the study cohorts that sum to high inter-individual variability often observed in the terms of response to cryostimulation.

Important variables, although hardly definable to the field, that can be the cause for the limited “biochemical evidence” in support of cryostimulation effectiveness, may be represented by the frequency and length of the treatment, the timing of application within the timing practice (e.g., training sessions), as well as subjective features that may alter the responsivity. For this reason, Lubkowska et al. (2011) demonstrated that the number of sessions importantly impacted the kinetics of secretion of biochemical markers of inflammation. Indeed, in 45 healthy men, five consecutive sessions of WBC increased IL-10 by 30% while 10 sessions determined a 17% decrease of IL-1α, 10% increase of IL-6, and 14% increase of IL-10. In both cases, the changes were no longer detectable after 2 weeks. On the contrary, 20 sessions of WBC had a similar impact on the cytokine profile, as for 10 sessions, but those effects were maintained 2 weeks after the end of the treatment. Other inflammatory mediators, such as IL-1β, TNFα, and IL-12 were unaffected by the treatment.

### WBC Can Diminish DOMS

The study of the preventive effects of WBC on DOMS was mostly demonstrated in the settings where specific exercise/training programs were applied. Compared with passive recovery, following four sessions of WBC (three-room device at −10, −60, and −110°C, with permanence in the therapy room for 3 min), the CRP increase was attenuated, and the anti-inflammatory IL-1 receptor antagonist (IL-1ra) was strongly induced in the runners who underwent a 45-min trail run specifically designed to induce the muscle damage. However, the exercise-associated changes in the TNF, IL-6, and IL-10 were unaffected. In addition, WBC led to a greater increase in the neutrophil count, compared with passive recovery, that may have accounted for a greater stimulus for angiogenesis that resulted in increased muscle perfusion and, hence, better recovery and lesser soreness (Pournot et al., 2011).

In physically active college-aged men who underwent an eccentric workout designed to induce DOMS, two-daily sessions of WBC over 5 days blunted the response of IL-1β and IL-6 while stimulating the secretion of IL-10 (Ziemann et al., 2014). Noteworthy, WBC reduced the physiological burden of an eccentric cycling bout and the rise of myoglobin and IL-15 concentrations, thus indicating the potential modulatory effects of WBC also on muscle strength (Jaworska et al., 2020). In a recent meta-analysis, Dupuy et al. (2018) reported that an acute cryostimulation exposure is a convenient tool to reduce DOMS. More recently, Poignard et al. (2020) found that cooling strategies, such as cryotherapy, is an effective strategy to reduce DOMS in professional tennis players. In this article, in comparison with cold-water immersion (CWI), WBC induced a greater feeling of recovery in this population. In line with this, Wilson et al. (2019) found that the WBC is a more effective strategy to recover from resistance training in comparison with CWI. However, the comparison of WBC with CWI in the management of DOMS still needs to be confirmed since several articles found no difference between WBC and CWI (Abaidia et al., 2016).

### WBC—PBC and Performance

All the following articles used in this Section are presented in Tables 1, 2 of the Section Cryostimulation Protocols.

**Table 1:** The studies investigating the acute effects of cryostimulation on the physical recovery.

**References**	**Outcomes** /**subjects**	**WBC or PBC treatment protocol**	**Cryostimulation effects compare to control condition (“+”: positive effect; “-”: negative effect; “=”: no change)**
Bouzigon et al. (2020)	Isometric muscle recovery Motocross riders 18 males	WBC (Aurore Concept, France) 1 exposure After training 30 s at −25°C and 3 min at −70°C with wind chill	+ Isometric strength - CMJ performance = Reaction time = Handgrip strength + Perceptive recovery
De Nardi et al. (2020)	The range of motion 11 young adult females	PBC (Criomed, Ukraine) 1 exposure 2 min 30 between −130 and −170°C	+ Range of motion
De Nardi et al. (2017)	Isometric strength Healthy adults 100 females 100 males	PBC (Criomed, Ukraine) 1 exposure 2 min 30 s between −130 and −160°C	+ Handgrip strength
De Nardi et al. (2015)	The range of motion Healthy adults 60 females 60 males	PBC (Criomed, Ukraine) 1 exposure 2 min 30 s between −130 and −140°C	+ Range of motion
Ferreira-Junior et al. (2014)	Neuromuscular performance Recreationally resistance-trained participants 13 males	PBC (Cryoness, Poland) 1 exposure 3 min at −110°C	= Peak torque = Average power = Total work = Muscle activity
Fonda and Sarabon (2013)	Muscle damage Healthy men 11 males	PBC Criomed 6 exposures 1 per day/6 days 3 min −140 to −195°C	+ Pain + knee flexion rate of torque development + Squat jump start power + Maximal torque production = CK concentration in blood + Heart rate variability (HRV) indices
Hausswirth et al. (2011)	Markers of muscle damage Well-trained runners 9 males	WBC (Zimmer Elektromedizin, Germany) 3 exposures at 1, 24, and 48 h post exercise −10°C, −60°C and 3 min at −110°C then 10 min seated comfortably in a temperate room (24°C)	Effect post 1 h/post 24 h/post 48 h +/+/+ MVC =/=/= CK concentration in blood +/+/+ perceived pain +/+/+ perceiced tiredness =/+/+ Well-being
Hohenauer et al. (2020)	Recovery after muscle damage Recreationally trained participants 28 females	PBC (Criomed, Ukraine) 1 exposure After training 30 s at −60°C and 2 min at −135°	- Muscular oxygenation = Arterial pressure + DOMS = Muscle swelling = MVIC = CMJ performance
Kruger et al. (2015)	Acute recovery Endurance athletes 11 males	WBC (Zimmer, Germany) 1 exposure After training −10°C, −60°C and 3 min at −110°C.	During subsequent exercise: + Maximal endurance performance + Cardio-respiratory parameters (VO_2_, HR) + Rating of perceived exertion + Muscular oxygenation
Piras et al. (2019)	Recovery during concurrent training Rugby players 9 males	PBC (Criomed, Ukraine) 1 exposure Between strength and endurance training 3 min at −160°C	During recovery: + HRV indices (SDNN, RMSSD, HF) + Baroreflex sensitivity During endurance training: + HR + VO_2_ + Minute ventilation + Energy cost = Respiratory exchange ratio = Blood lactate concentration = Net energy expenditure derived from aerobic energy sources
Russell et al. (2016)	Physiological performance and perceptual responses English Premier League academy soccer players 14 males	WBC (Juka, Poland) 1 exposure After training 30 s at −60°C and 2 min at −135°C	= CMJ performance = Blood lactate concentration = CK concentration in blood + Salivary testosterone concentration = Salivary cortisol concentration
			= Perceived soreness = Perceived recovery
Vieira et al. (2015)	Vertical jump recovery Resistance-trained participants 12 males	PBC (Cryoness, Poland) 1 exposure After training 3 min at −110°C	= Muscle power
Wilson et al. (2019)	Recovery after resistance training Resistance-trained participants 24 males	WBC (Juka Cryotherapy Chamber) 2 exposures After training 3 min at −85°C, 15 min warming period and 4 min at −85°C	Effects post 24 h / post 48 h / post 72 h: = / - / = DALDA score + / = / = Perceived soreness - / = / = Peak torque = / = / = MVIC = / - / - Reactive strength - / = / - CMJ performance = / + / = Isometric peak force - / + / = RFD = / - / - CK concentration in blood = IL-6 concentration in blood = / - / - CRP concentration in blood = / - / = TNF-α concentration in blood
Wilson et al. (2017)	Recovery following a marathon Endurance athletes 31 males	WBC (Mecotec, Germany) 2 exposures After marathon 3 min at – 85°C, 15 min warming period and 4 min at−85°C	Post 24 h / post 48 h effects: = / = DALDA score = / + Perceived soreness - / - Muscle function (peak torque, reactive strength index) - / = CK concentration in blood - / - IL-6 concentration in blood - / + CRP concentration in blood = / = TNF-α concentration in blood

*WBC, whole-body cryotherapy; PBC, partial-body cryotherapy; DALDA, The Daily Analysis of Life Demands for Athletes; DOMS, delayed onset of muscle soreness; CMJ, countermovement jump; MVIC, maximal voluntary isometric contraction; RFD, rate of force development; HR, heart rate; HRV, heart rate variability; RMSSD, root mean square standard deviation; SDNN, standard deviation of the NN (R–R) intervals; HF, high-frequency power; LF, Low-frequency power; VO_2_, Oxygen consumption; VO_2_ max, Maximal oxygen consumption; CK, creatine kinase; IL-6, interleukin 6; IL-15, interleukin 15; CRP, C reactive protein; TNF- α, tumor necrosis factor-α; BDNF, brain-derived neurotrophic factor; IGF1, insulin-like growth factor*.

**Table 2:** The studies investigating the chronic effects of cryostimulation on the physical recovery.

**References**	**Outcomes** **/subjects**	**WBC or PBC treatment protocol**	**Cryostimulation effects compare to control condition (“+”: positive effect; “-”: negative effect; “=”: no change)**
Broatch et al. (2019)	Physiological and performance adaptations Recreational athletes (triathlon or cycling) 22 males	WBC (Zimmer, Germany) 12 exposures After training −10°C, −60°C and 3 min at – 110°C	= Maximal aerobic power = VO_2_ max = Time to exhaustion = Performance in the time trial = Blood markers (adrenaline, noradrenaline, cortisol) = Sleep quality (time in bed, sleep duration, sleep latency sleep efficiency …)
Jaworska et al. (2021)	Growth factors concentrations, amino acids profile and motor abilities in professional judokas	WBC (Zimmer, Germany) 10 exposures – One a day 2 h after training 30 s at −60°C and 3 min at −110°C	+ Circulating levels of two growth factors (BDNF and IGF-1) + Amino acid profile + Specific judo abilities + Muscle function
Jaworska et al. (2020)	Resistance training supported by cryostimulation Untrained students 17 females 13 males	WBC (Unknown model) 12 exposures during 4 weeks of resistance training 30 s at – 60°C and 3 min at −110°C	+ Isokinetic muscle strength + Pedal force + Myoglobin concentration in blood + Blood marker concentration (myostatin, IL-15) + Muscle pain
Jaworska et al. (2018)	Specific training supported by cryostimulation University volleyball players 10 females 10 males	WBC (Unknown model) 10 exposures during 2 weeks of specific volleyball training 30 s at −60°C and 3 min at −110°C	+ Limitation of physical performance decrease + Concentration of growth factors (BDNF, IGF1) in blood
Klimek et al. (2010)	Aerobic capacity and maximal anaerobic power Students of physical education 15 females 15 males	WBC (Unknown model) 10 exposures −60°C and 3 min at −110°C	= Aerobic capacity + Maximal anaerobic power
Le Meur et al. (2017)	Maximal incremental running test 16 triathletes in functional overreaching	WBC Zimmer 7 exposures on 7 days during 1 week of tapering	+ Performance in maximal incremental test = Change in perceived fatigue with CONT
Lubkowska and Szygula (2010)	Aerobic capacity 25 healthy males	WBC (Unknown model) 15 exposures 30 s at −60°C and 3 min at −130°C	= VO_2_max - Red Blood Cell concentration in blood - Hemoglobin concentration

*WBC, whole-body cryotherapy; PBC, partial-body cryotherapy; DALDA, The Daily Analysis of Life Demands for Athletes; DOMS, delayed onset of muscle soreness; CMJ, countermovement jump; MVIC, maximal voluntary isometric contraction; RFD, rate of force development; HR, heart rate; HRV, heart rate variability; RMSSD, root mean square standard deviation; SDNN, standard deviation of the NN (R–R) intervals; HF, high-frequency power; LF, Low-frequency power; VO_2_, Oxygen consumption; VO_2_ max, Maximal oxygen consumption; CK, creatine kinase; IL-6, interleukin 6; IL-15, interleukin 15; CRP, C reactive protein; TNF- α, tumor necrosis factor-α; BDNF, brain-derived neurotrophic factor; IGF1, insulin-like growth factor*.

In the past decades, cryostimulation was often used by athletes, trainers, and sport physicians to promote enhanced athletic performance and recovery (Banfi et al., 2010; Lombardi et al., 2017). However, in high-performance sport, the question remains if cryostimulation can or should be used before an event to enhance the performance of an individual and the physical well-being or only to improve the recovery parameters in the athletes.

Current evidence supporting its utilization is limited and contradictory: the testimony of athletes is generally positive but it could be influenced by a possible placebo effect. From what can be deduct from testimonies of coaches and athletes, it seems that exposure to cryostimulation prior than ~3 h to competition could have a performance enhancement effect (Partridge et al., 2019). The use of cryostimulation before training or competition may have a beneficial effect by a multi-factorial hypothesis, such as the positive effects of hormonal changes (Hornery et al., 2005; Rose et al., 2017), with an increase of circulating cortisol and testosterone, and by a peripheral vasoconstriction, which consequently leads to high muscle oxygenation afterward (Hornery et al., 2005), and by a psychological well-being. The scientific community will have to study the possible effects of cryostimulation on sport performance, since further studies are required to provide recommendations for the coaches and athletes based on evidence. In fact, only few studies examined this topic.

Studying physical performance, Ziemann et al. (2012) found improvements in tennis drills execution after a cycle of 10 WBC exposures (3 min; −120°C) carried out for 5 consecutive days (two times a day) in 12 high-ranking professional tennis players, demonstrated by a 7% improvement in stroke effectiveness, measured through shot accuracy and speed. Schaal et al. (2014) found, in 10 elite synchronized swimmers, a reduction of swimming speed in a 400 m trial between before and after an overreaching period in the experimental group after a cycle of 10 sessions of WBC exposures (3 min; −110°C) compared with the control group. Further studies, maybe without methodological differences in terms of exposure temperature, duration, and sessions dose, are required to confirm the cryostimulation effectiveness in sport specific performances, such as tennis stroke accuracy or swimming speed.

Le Meur et al. (2017) have studied the effect of 1 week WBC exposures (3 min; −110°C) in 16 functionally overreached triathletes. The results showed that completing multi exposures to WBC induced a larger performance supercompensation after a simulated 1-week taper in the functionally overreached endurance athletes. These preliminary findings showed that multiple exposures to WBC after a period of heavy training may mitigate the signs of accumulated fatigue during the intensified training blocks.

#### Aerobic and Anaerobic Performances

In the scientific literature, only few studies evaluated the influence of cryostimulation on the aerobic adaptation parameters. Klimek et al. (2010) enrolled 30 subjects (15 men and 15 women) to assess the influence of WBC on aerobic and anaerobic capacities. The participants underwent two ergocycle trials before and after a cycle of 10 consecutive WBC sessions. The authors calculated baseline aerobic capacity by means of a progressive cycle ergometer test and the anaerobic power by performing a 20-s Wingate test. After finishing the 10 WBC sessions, there were no changes in the aerobic capacity for both genders. Only men showed an increase in the maximal anaerobic power and capacity after 10 sessions in a cryogenic chamber. The authors concluded that in sports disciplines with a predominance of anaerobic metabolism, it could be advisable to introduce WBC treatment in the training periodization, at least in a male population. However, the lack of a control group could be a limit of the aforementioned study.

The same group of authors evaluated (Klimek et al., 2011) the effects of a single WBC treatment on the dynamics and the level of maximal aerobic power, enrolling 30 subjects (15 women and 15 men) and applying a Wingate test after each WBC session (six consecutive treatments at −130°C) in the 15, 30, 45, 60, 75, and 90th min after leaving the cryo-chamber. They found that a single WBC treatment may have a minor influence on the short-term anaerobic performance without significant changes in both genders but leads to the improvement of velocity during the start as expressed by a shortened time to obtain the maximal anaerobic power.

An interesting study (Kruger et al., 2015) showed that WBC improves acute recovery during high-intensity intermittent exercise in thermoneutral conditions. In a randomized crossover trial, 11 endurance-trained male athletes performed two ramp-test protocols to individual exhaustion within an hour, interspersed with a high intensity running protocol, consisting of 5 ×5 min at 90% of maximum velocity with 4 min of active recovery between the intervals. During the recovery period, which lasted 1 h, the subjects were randomly assigned to WBC (3 min, −110°C) or control condition (3 min slow walk). The authors found that the difference in the time from the beginning of the ramp tests to the individual exhaustion of the participants was significantly lower during WBC intervention than in the control group. In addition, the rating of perceived exertion (RPE) values was lower at submaximal intensities, as well as for oxygenation of the vastus lateralis muscle, heart rate, and peak oxygen uptake (VO_2peak_) values.

On the other hand, Broatch et al. (2019) involved 22 well-trained men in a two-group parallel research where the participants performed 4 weeks of cycling high-intensity interval training (HIT), with each training session followed by a 3 min of WBC exposure (−110°C) or to passive control. The authors measured basal adrenal hormones changes, sleep patterns, and performance tests, such as a graded exercise test, a time to-exhaustion test, a 20-km time trial, and a 120-min submaximal test. The statistical analysis did not show significant effects of WBC on the performance parameters, suggesting that a regular post-exercise cryostimulation is not an effective strategy to increase the training-induced aerobic adaptations to 4 weeks of HIT. Considering this information, further studies are required to elucidate the influence of cryostimulation on aerobic and anaerobic mechanisms.

#### Strength

Despite the increasing popularity of cryostimulation in sport medicine, very few studies have investigated the acute or long-term effects of very-low temperatures on the muscle strength performance, while, on the contrary, there are significantly more studies on the influence of cryostimulation as a recovery technique after strength exercises (Rose et al., 2017).

In a very recent article, Jaworska et al. (2020) found an improvement in the average power and isokinetic extension muscle strength when combining cryostimulation with resistance training. Twenty-five volunteers completed a 4-week protocol which included 12 sessions of resistance training lasting ~50 min, focused on the lower limbs, each session followed either by WBC exposure (3 min, −120°C) or passive recovery. They observed that training combined with WBC induced a significant increase of maximal average power in the knee isokinetic extension strength test, while in the control group, the level of the aforementioned test remained unchanged. These changes were accompanied by a drop in the myostatin and IL-15 concentration in the experimental group. In the current study, the authors did not record changes in muscle mass, therefore, they stated that the increase of isokinetic muscle strength could be connected to a better motor unit recruitment.

In another study, a single session of PBC (3 min, −110°C) was used to evaluate the effects of cryostimulation on the elbow's flexor neuromuscular performance by Ferreira-Junior et al. (2014). They enrolled 13 subjects and exposed them to two different experimental conditions separated by 72 h: a single session of PBC and a control condition (3 min, 21°C). The protocol consisted of a maximal isokinetic elbow flexion test repeated 30 min before and 10 min after each condition. The authors did not find significant differences in peak torque, average power, total work, nor muscle activity between the conditions, suggesting that cryostimulation could be utilized before training or rehabilitation without compromising the neuromuscular performance of the elbow flexors.

In addition, Westerlund et al. (2009) studied the effects of single and repeated WBC sessions (2 min, −110°C) on neuromuscular performances in healthy subjects. The authors enrolled 14 participants and administered the WBC sessions three times a week for 3 months. The neuromuscular performance tests included a drop-jump exercise and a maximal voluntary contraction force of the wrist flexors which were performed before and after the WBC at the beginning (a single WBC session) and at the end of the 3-month study period (repeated WBC sessions). After a single very-low temperature exposure, the flight time decreased significantly, however after repeated WBC, only a similar tendency was found. This adaptation was accompanied by a decreased co-contraction of lower leg muscles during the drop-jump. The maximal force level did not change significantly, either after a single or after repeated WBC sessions. The authors stated that, concerning dynamic exercises, neuromuscular functioning may be able to adapt to repeated WBC exposures.

Concerning isometric strength, Costello et al. (2012) enrolled 36 volunteers in a cross-over study, which included a WBC session (3 min, −110°C) and control condition (3 min, 15°C). All subjects were exposed to both treatments after a lapse of 2 h. They were asked to complete maximal voluntary isometric contraction (MVIC) of the right knee extensors. The baseline tests were completed immediately before the WBC session and post-tests and followed immediately after each exposure and again 15 min later for both of the temperature conditions. MVIC was recorded in a sitting position: the participants were asked to maximally contract their right leg for 3 s. They completed the protocol three times and the maximum value of MVIC was recorded. The authors did not find significant differences in MVIC between the groups following treatment suggesting that the WBC treatment did not affect MVIC of the knee extensors.

De Nardi et al. (2017) reported an improvement in the concentric hand-grip strength after PBC. Enrolling a consistent number of participants (200 healthy adults divided both in a PBC (150 s, −130/−160°C) and in the control group (150 s, 22°C), the authors administered a baseline handgrip strength test before each condition. Immediately after the exposure in the cabin, both groups performed a subsequent handgrip test. Data showed that both the groups exhibited an increase in their handgrip strength values, especially the PBC group. The authors concluded that PBC could also be performed before a training session or a sports event.

The results mentioned above show that WBC and PBC exposures are not deleterious in strength performance. WBC exposures performed after resistance training could even have positive effects on the strength development through a better motor unit recruitment.

#### Flexibility

A variety of studies (Ma et al., 2013; Giemza et al., 2015; Romanowski and Straburzynska-Lupa, 2020) have found favorable outcomes in the improvement of range of motion (ROM) and flexibility after one or multiple cryo-exposures. Ma et al. (2013) studied the effects of a cycle of 24 WBC sessions (3 min, −110°C) on the active ROM of flexion, abduction, internal, and external rotation of the shoulder in subjects suffering from adhesive capsulitis of the shoulder. The authors divided 30 patients into two groups: the WBC group received physical therapy modalities, passive joint mobilization of the shoulder, and cryostimulation, whereas the control group received only physical therapy modalities and passive join mobilization of the shoulder. Each group showed a significant improvement in the ROM measures from baseline to the end of the study, with the WBC group showing a significant difference for active ROM of flexion, abduction, internal rotation, and external rotation compared with the controls.

On the other hand, Romanowski and Straburzynska-Lupa (2020) evaluated the beneficial supplement to exercise therapy of cryostimulation on functional parameters, such as spine mobility and chest expandability in patients with ankylosing spondylitis. Curiously, they divided 92 patients in the three groups, evaluating the effects of two different WBC modalities (−60 and −110°C) combined with an exercise therapy and respectively with exercise therapy alone. After an 8-day period, they demonstrated that the −110°C WBC group manifested a significant improvement in spine mobility and chest expandability.

Giemza et al. (2015) evaluated the effects of frequent WBC (3 min, −120°) treatments on back pain therapy in elderly men, focusing their attention on the lumbo-thoracic spine mobility, measuring active flexion and extension, rotation to the right and the left, and lateral flexion to the right and the left. The patients who exercised and underwent WBC five times a week showed a significant increase in the range of the lumbar spine mobility.

De Nardi et al. (2015) studied the acute effects of a single session of PBC (150 s, −130/−160°C) on sit-and-reach amplitude in a population of both genders, enrolling 60 men and 60 women. Male and female were in turn randomly divided into two groups: the PBC group and the control group (150 s, 21°C) and after an initial sit-and-reach test, they were exposed to the experimental condition. Immediately after the experimental session, both groups performed another sit-and-reach test. The results showed that the PBC group improved their sit-and-reach amplitude to a greater extent than the control group, leading to the conclusion that the ROM is increased immediately after a single session of cryostimulation.

In a very recent paper, the same group of authors (De Nardi et al., 2020), studied the impact of different sessions of PBC (150 s, −130/−160°C) on trunk- and lower limbs flexibility in a sample of young women. To evaluate the flexibility responses to the interventions, they proposed three tests: standing hamstring, stretch test, sit-and-reach test, and active knee extension test. For this study, 11 healthy women were enrolled and were randomly subjected to the four conditions (hamstring stretching in an upright position; hamstring stretching in an upright position in conjunction with whole-body vibration; PBC; and rest, alias sitting position). All of the aforementioned dependent variables were measured before and immediately after each experimental condition. Concerning active knee extension, the outcomes revealed a significant improvement in ROM after PBC in comparison to the control.

Even if each aforementioned study on very-low temperatures exposures lead to a positive effect on the flexibility and ROM, we agree with Bleakley and Costello (2012), highlighting that further studies are needed to determine the *in vivo* effect of WBC/PBC on ROM, flexibility and tissue stiffness, splitting, among the other issues, between exposure to wet and dry cold, which is the typical condition of WBC/PBC.

#### WBC—PBC and Sleep

Sleep is probably one of the most important phases in the recovery of an athlete. Unfortunately, many elite athletes suffer from sleep problems and the accumulated risk of sleep debt can lead to the risk of injury and increase the prevalence of overtraining (Lastella et al., 2018; Sargent et al., 2021). It has been proposed that cryostimulation may lead to improved sleep. Currently, there are still few studies on the subject, however the existing data is encouraging. Indeed, Bouzigon et al. (2014) report that exposure to cryostimulation improves the perception of sleep in high-level basketball players. This finding was also reported by Schaal et al. who showed that repeated cryostimulation exposure during a phase of intense training improves the perception of sleep in a group of female swimmers (Schaal et al., 2014). In addition to these perceptive data, Douzi et al. reported that exposure to cryostimulation (immediately after a training session) is associated with a reduction in the number of movements during the night (measured through actigraphy), which could mean a better quality of sleep (Douzi et al., 2018, 2019). All these results are encouraging but need to be confirmed.

## Cryostimulation: FOE or Friend

The use of cold application in the context of sports recovery has received special attention and several studies have drawn attention to the fact that exposure to cold can be harmful to the training adaptations and therefore lead to negative consequences on physical performance. These studies were mainly carried out with cold baths and examined whether the repeated use of cooling applications after endurance training and resistance training influenced physical performance and molecular and morphological adaptations of skeletal muscle. For further details, we encourage reading the excellent review by Hyldahl and Peake (2020). Currently, the use of repeated cold applications appears to affect the strength and/or aerobic performance underlying that the physiological adaptations may occur in different ways. Indeed, Malta et al. (2021) very recently reported that exposure to repeated cold could lead to negative consequences on strength performance but not on aerobic performance. A review by Ihsan et al. (2021) showed that a “recovery periodization” using cold therapies may be of importance to improve the performances of athletes. Nevertheless, the authors advised to avoid CWI following a training focused on improving the muscle strength or hypertrophy.

The available data on the potential consequences of repeated exposure to cold on the physiological adaptations to aerobic training appear to show no negative effects on aerobic performance. Only one study to date has shown that CWI in six sedentary subjects reduced adaptations to aerobic training. The cold exposure in this study consisted of 20 min at 5°C, four times per week. This protocol seems to be in contrast with the recommendations of Machado et al. (2016) who proposed immersion in cold water for 10–15 min at 10–15°C. Later, the studies have shown non-damaging effects of cold exposure on aerobic performance (Halson et al., 2014; Broatch et al., 2019). The studies have sometimes reported that the cold can lead to better muscle perfusions and even mitochondrial biogenesis (Hyldahl and Peake, 2020). However, these results need to be confirmed. Regarding cryostimulation, only Broatch et al. (2019) reported no effect of cryotherapy on the increase in VO_2_max. To date, the exposure to cold does not seem to be contraindicated for aerobic performance and seems to be suitable in a case of important fatigue.

Concerning the impact of cold exposure on the physiological adaptations of muscular training, Yamane et al. (2006) provided the first evidence that repeated immersion in cold water after resistance training attenuated grip strength gains while not influencing changes in muscle endurance. Since this study, several studies have confirmed the risk of using cold exposure after strength training (Hyldahl and Peake, 2020). Malta et al. (2021) in their recent meta-analysis confirmed this finding. This decrease in muscle performance may be associated with a negative effect of cold on muscle hypertrophy and protein synthesis. However, as Hyldahl and Peake (2020) suggested, these findings generally indicate that regular cooling attenuates chronic gains in strength following traditional resistance training, whereas it has no influence on short-term adaptation after muscle-damaging exercise. It has even been shown that during weeks with an intense training load, the cold immersion can reduce the risks of maladaptation to training (Tavares et al., 2019). These cold baths might even be recommended in the phases of intense training where there is not enough time to recover. It is therefore important to periodize the use of cold in recovery according to the objectives. For example, during periods of significant muscular work aimed at hypertrophy, cold is not recommended. However, during periods of overload training, during periods of tapper or competition, it may be recommended. However, one needs to keep in mind that all these studies were conducted using the cold baths.

Nevertheless, a very recent study (Jaworska et al., 2021) showed that the use of WBC daily after exercise training in 13 Judokas of the National Judo Team of Poland during a 10-day traineeship did not alter muscle performance, sustained muscle function, and slightly improved specific judo abilities. The authors explained the differences with the results shown with the cold bath by the duration between the end of the exercises and the exposures (2 h for the cryo vs. immediately after the cold bath). Another very recent study demonstrated that the combination of 12 resistance training sessions with WBC induced a positive and likely significant improvement of isokinetic muscle strength. The authors showed that the resistance training combined with cold exposure modified the muscle strength through the modulation of myostatin and IL-15 concentrations (Jaworska et al., 2020).

Some investigations are required to increase the knowledge concerning the effect of cryostimulation exposure on muscular function and recovery. It is not relevant to draw general conclusions such as “it works” or “it does not work.” The cryostimulation can be efficient in some aspects and not in others.

## Indications—Contraindications of WBC—PBC

Cryostimulation application must adhere to the precise guidelines and indications. There are some contraindications which can limit its field of applications (Lombardi et al., 2017; as shown in the Appendix A). However, it should be underlined that the indications and contraindications come from the empirical application of the treatment and theoretical precautions (e.g., the potential deleterious effects of cold in cardiovascular diseases), rather than from the established guidelines that are still lacking, as well as approval from the national and supranational regulatory agencies. Some safety concerns exist, as partially revised by Costello et al. (2015). Indeed, while WBC is generally performed in cryochambers labeled as medical devices, the safety concerns are limited and mainly account for the patient-related reasons that escaped the anamnesis of physicians, PBC is mainly performed in non-medical centers with non-medical devices and, hence, safety rules are less stringent. Further, the direct exposure to liquid nitrogen vapors is related to the potential risk of asphyxia. The cold burns and cold urticaria represent the most common adverse events. However, some rare cases with significant health problems have been reported during and after the exposure (Camara-Lemarroy et al., 2017; Carrard et al., 2017; Greenwald et al., 2018).

In general, cryostimulation must be performed in controlled conditions in the presence of specifically formed personnel. WBC is considered to be a safe procedure and no deleterious effects have been reported neither for the lungs (Smolander et al., 2006), regardless of the inspiration of cold air, nor for the heart (Banfi et al., 2009a). Precaution might be taken regarding the treatment of subjects affected by cardiovascular diseases since the previous studies reported a slight, although clinically irrelevant increase in systolic blood pressure (Lubkowska and Szygula, 2010). PBC needs more attention and control because the individuals are in direct contact with nitrogen. Nitrogen should not be inspired, and the direct nitrogen jet increases the risk of frostbite. Consequently, cryostimulation is not indicated for the patients with unstable angina pectoris, cardiac failure in III and IV stages according to the New York Heart Association (NYHA), the United States (Lombardi et al., 2017).

## Cryostimulation Protocols: Procedure Suggestions and Recommendations

In the case of a new subject wishing to use cryostimulation exposure, an intake consultation is highly recommended. An intake consultation is the first interaction and consists of an intake interview and intake session. An intake interview should be designed to address the purpose and needs of the users, exclude the absolute- and relative contraindications (Appendix A), determine the profiles of the users, and assess the preferences of users. An intake session, a first mild-dosage session, provides the operator information on how the user responds to the cryostimulation and helps to get acquainted. At the end of an intake session, the treatment settings and cycle should be recommended.

To ensure effective, repeatable, and standardized effects, the application of cryostimulation entails a fixed procedure tailored to the needs and personal profile of an individual. As there is no one-size-fits-all solution, and there are many “moving parts” in this decision-making, it is crucial to gather feedback, observe progress, and adjust throughout the treatment cycle.

Recommendations:

1. Although cryostimulation is a safe treatment with no reported adverse side effects in proper usage, it may have health risks in certain medical conditions. Therefore, it is imperative to exclude the absolute and relative contraindications before each session, as the health condition of users is a safety and liability issue.
2. The cryo-operator should explain the methodology and safety precautions to each person before each session, as the person changes into the proper safety attire consisting of headwear, footwear, a mask, and gloves.
3. The type, severity, and stage of the symptoms should determine the cold dose; more acute, severe symptoms require more significant skin temperature drops and/or more regular exposures. Too little cryo exposure will not produce the desired results, while too much may become harmful.
4. Cryostimulation should not be a one-size-fits-all approach. A one-size-fits-all approach will be less safe, is considered one of the reasons for the lack of consistency and significant benefits in the research, and creates a considerable variation in how cryostimulation is perceived. Multiple studies illustrate significant differences in the outcomes based on the personal profile differences, such as body composition, gender, training status, age, skin type, and responsiveness (Cholewka et al., 2012; Hammond et al., 2014; Cuttell et al., 2017; Polidori et al., 2020; Kujawski et al., 2021). For instance, women cool down quicker than men and therefore require a milder cold dose. Different approaches are recently validated (Polidori et al., 2016; Broede et al., 2017) using the thermophysiological models that take these personal differences into account and standardize the effects.
5. A fixed number of sessions within a constrained time frame to obtain optimal benefits of optimal individuals is recommended (as shown in Section Cryostimulation Protocols). For instance, after intense activity, the cycle for performance recovery purposes should contain 1–5 sessions, with at least two sessions within 24 h. On the other hand, the cycle for treating moderate, rheumatoid arthritis complaints requires 20 consecutive sessions, one session per day for 4 weeks.
6. Habituation throughout a cycle is evident and should be acknowledged (Louis et al., 2020). In case of occurrence, the thermal shock will be less amplified (Yurkevicius et al., 2021). Consider increasing the treatment dose over time to prevent this from happening.
7. Real-time monitoring of the physiological and thermal responses combined with personal feedback will further optimize the complete WBC approach. For example, one could assess the thermal profile of individuals by taking pre- and post-blood pressure (i.e., the parasympathetic response), pre-and post-skin temperature measurements, and considering the reactions and susceptibility of individuals, such as shivering, cold pain, body language during the exposure, and the thermal comfort of the clients.
8. The treatment cycle should be fitted within the existing regime of the indication, whole-body cryo should not be considered as an act on its own.

## Conclusion

This position/consensus work should be seen as a companion paper of Bouzigon et al. (2016), Lombardi et al. (2017), Dupuy et al. (2018), and the information note published by the International Institute of Refrigeration (Dugué et al., 2020), but here focused on the potential use of WBC and PBC for recovery purposes after exercising in the athletes. The review globally confirms the interest in the use of the cryostimulation in sport field. However, as expressed in our recently published information note, more information and data should be available to fully justify and understand the benefits of cryostimulation. The diversity of the cold apparatus that is used as well as the protocol discrepancies and the very different circumstances in which data have been collected retain to provide good robust evidence of the benefits. There are several issues, such as the dose effect/treatments to optimize the protocols (depending on the exposure, the desired effect, and the characteristics of the subject) as well as safety issues that should be tackle.

In Pubmed, the number of published works concerning WBC is over 700 and the number related to the sub-topic of recovery of athletes is <60. These numbers are globally quite low but interestingly, in the latter case, more than one-third of the articles have been published in the past 3 years reflecting a growing understanding of the importance of WBC in the recovery process of the athletes.

## Author Contributions

RB, OD, and BD contributed to manuscript revision. RB, OD, BD, IT, MD, J-PB, TM, DT, EM, and GL wrote sections of the manuscript. All authors contributed to manuscript revision, read, and approved the submitted version.

## Conflict of Interest

IT was employed by company ProCcare BVBA and Mobilito Sport. MD was employed by company Krioplanet Ltd. J-PB was employed by company Air Liquide Group International. The remaining authors declare that the research was conducted in the absence of any commercial or financial relationships that could be construed as a potential conflict of interest.

## Publisher's Note

All claims expressed in this article are solely those of the authors and do not necessarily represent those of their affiliated organizations, or those of the publisher, the editors and the reviewers. Any product that may be evaluated in this article, or claim that may be made by its manufacturer, is not guaranteed or endorsed by the publisher.
