# Supplementary material for: Cryostimulation for Post-exercise Recovery in Athletes: A Consensus and Position Paper
Source: Front Sports Act Living. 2021 Nov 24;3:688828. doi: 10.3389/fspor.2021.688828 (PMC8652002; doi:10.3389/fspor.2021.688828)
Supplement: Supplementary file 1 [file Data_Sheet_1.pdf]

Appendix A

Checklist absolute and relative contraindications

Absolute contraindications

The subject should NOT undergo whole-body cryo. A factor or condition present precludes exposure to whole-body cryo; if performed, it might harm the client and is regarded as a negligent act.

**Absolute contraindications**	**Yes or No**
1. Untreated high blood pressure	
2. heart attack within the past six months	
3. Decompensated diseases of the cardiovascular and respiratory system	
4. Unstable angina	
5. Pacemaker	
6. Peripheral artery occlusive disease (Fontaine stages III and IV)	
7. History of deep vein thrombosis	
8. Acute febrile diseases of the respiratory tract	
9. Acute renal and urinary disorders	
10. Severe anemia	
11. Signs or symptoms of cold allergy	
12. Severe wasting diseases	
13. Seizure disorders	
14. Large area bacterial and viral skin infections wound healing problems	
15. Alcohol and drug influence	

Relative contraindications

Caution should be used if one of these conditions is met. In case one condition is met, it is acceptable if the benefits outweigh the risks, although the personal doctor should be consulted. In case more than one condition is met, this situation should be regarded as an absolute contra-indication.

**Relative contraindications**	**Yes or No**
1. Cardiac arrhythmias	
2. Heart valve defects	
3. Status post heart surgery	
4. Ischemic heart disease	
5. Raynaud's syndrome	
6. Polyneuropathies	
7. Pregnancy, starting in the 4th month	

8. Vasculitis	
9. Claustrophobia	
10. Hypothyroidism	
11. Hyperhidrosis	

Disclaimer:

The list of absolute- and relative contraindications presented above is not an exhaustive list and should not be regarded as one. It is merely a guideline based on the available literature. If either the practitioner or client has any concerns regarding an individual's medical history, it is essential to seek a medical opinion before exposure, as safety is paramount.

Precautions

- Be sure that the client enters the unit completely dry, wearing shorts/bikini or tank top
- Be sure that the client does not wear wet socks or any other wet clothing.
- Be sure that the client does not wear: make-up, lotion, or moisturizing crème.
- Be sure that the client does not wear a hearing assistance device.
- Be sure that the client does not wear: jewelry, piercing, contact lenses, glasses, or other metal items.
- Be sure that the client does wear personal protection equipment for sensitive areas: gloves, ear protection, closed slippers.
- Be sure that the client leaves the cabin when instructed.

Please instruct the client that in case he would experience any pain or discomfort during the whole-body cryo session, he should leave the unit and end the session immediate
